# Motor development and motor resonance difficulties in autism: relevance to early intervention for language and communication skills

**DOI:** 10.3389/fnint.2013.00030

**Published:** 2013-04-24

**Authors:** Joseph P. McCleery, Natasha A. Elliott, Dimitrios S. Sampanis, Chrysi A. Stefanidou

**Affiliations:** School of Psychology, University of BirminghamWest Midlands, Birmingham, UK

**Keywords:** autism, motor, early intervention, communication, language

## Abstract

Research suggests that a sub-set of children with autism experience notable difficulties and delays in motor skills development, and that a large percentage of children with autism experience deficits in motor resonance. These motor-related deficiencies, which evidence suggests are present from a very early age, are likely to negatively affect social-communicative and language development in this population. Here, we review evidence for delayed, impaired, and atypical motor development in infants and children with autism. We then carefully review and examine the current language and communication-based intervention research that is relevant to motor and motor resonance (i.e., neural “mirroring” mechanisms activated when we observe the actions of others) deficits in children with autism. Finally, we describe research needs and future directions and developments for early interventions aimed at addressing the speech/language and social-communication development difficulties in autism from a motor-related perspective.

## Introduction

Autism is a pervasive developmental disorder that is diagnosed based upon behavioral criteria for impairments in social skills, communication and language skills, and restricted interests and repetitive behaviors. Autism is currently considered to be a “spectrum” disorder, with three Pervasive Developmental Disorders now being termed Autism Spectrum Disorders (ASDs): Autistic Disorder, Aspergers Disorder, and Pervasive Developmental Disorder-Not Otherwise Specified (PDD-NOS). Individuals with these three different ASDs differ somewhat in regards to the nature and/or severity of their early language and intellectual difficulties. However, individuals with these three ASDs are similar in that they share impairments in social and communication skills, and that the onset of their difficulties begins by three years of age (American Psychiatric Association, 2000).

The only motor abnormalities currently included in the diagnostic criteria for ASDs are stereotypical repetitive behaviors (American Psychiatric Association, 2000; see also Lord and Jones, 2012). These repetitive behaviors include motor stereotypies, such as hand and finger mannerisms, body rocking, and arm flapping (Lord et al., 1994; Loftin et al., 2008). However, impairments in motor development commonly observed in children and adults with ASDs are not limited to motor stereotypies (Kopp et al., 2010; Linkenauger et al., 2012). Early motor delays, gait abnormalities, and difficulties with gross and fine motor coordination, postural control, and imitation have been found to constitute significant neurological co-morbid conditions in this population (Provost et al., 2007; Bhat et al., 2011; Maski et al., 2011). For example, Nobile et al. (2011) examined motor dysfunction in ASDs and found that children diagnosed with Autistic Disorder presented with stiffer gait, difficulties maintaining a straight line while walking, and postural abnormalities. Similarly, other studies have reported an “ataxic” gait in adults with autism (Hallett et al., 1993), and reduced postural stability, especially when somatosensory input was disrupted (Minshew et al., 2004). Deficits in postural stability and motor coordination in individuals with ASDs were confirmed through a recent meta-analysis conducted by Fournier and colleagues (Fournier et al., 2010). Children and adults with autism have also been found to exhibit praxis and imitation difficulties, including manual, postural, and orofacial imitation (Rogers et al., 1996, 2003; Stone et al., 1997; Stone and Yoder, 2001; Williams et al., 2004; Mostofsky et al., 2006; Dziuk et al., 2007; Vanvuchelen et al., 2007, 2010; Stieglitz Ham et al., 2008; Dowell et al., 2009). Critically, evidence suggests that deficits in motor skills, coordination, and balance are not limited to individuals with ASD experiencing cognitive delays (Jansiewicz et al., 2006). A variety of mechanisms have been proposed to account for the motor functioning differences observed in individuals with ASDs, including abnormalities in the cerebellum (Fatemi et al., 2012), impairments in frontal-striatal connections (Fournier et al., 2010), difficulties in self-other mapping (Williams et al., 2001), impaired sensory input (Gowen and Hamilton, 2013), and impaired multisensory integration (Gowen and Hamilton, 2013).

The aim of the current review is to outline the evidence for ASD-related motor development and motor resonance difficulties, and to examine current research on interventions that attempt to apply motor-related approaches to improve speech/language and social communication skills in children with autism. Similar to recent reviews by others (e.g., Iverson, 2010; Bhat et al., 2011), we first describe the existing evidence for early delayed, impaired, and atypical motor development in autism. In this review, we place particular emphasis on research related to several motor development mechanisms and milestones believed to be associated with concurrent and later speech/language and social communicative functioning. Next, we address current evidence for impairments in motor resonance (i.e., “mirror neuron”) functioning in individuals with autism, which has implications for social engagement during communication interactions. After this, we carefully examine and evaluate the existing motor-related autism intervention research that targets speech/language and social-communication skills. This includes augmentative and alternative communication (AAC) interventions, more directly motor-based behavioral interventions, electromagnetic brain stimulation interventions, and interventions that utilize synchronous motor activities to increase speech/language and social communication skills. The current review differs distinctly from previous reviews, which have focused primarily on interventions for sensorimotor skills themselves (e.g., Baranek et al., 2008; Bhat et al., 2011), as opposed to motor-related attempts to specifically target speech/language and communication skills. We conclude our review by describing research needs and future directions for research on early interventions for speech/language and social-communication skills from a motor-related perspective.

## Early motor development in autism

Evidence suggests that autism is caused by a complex combination of multiple genetic and environmental factors. Twin studies examining the concordance of autism in monozygotic versus dizygotic twins provide evidence that genetics play a key role (Folstein and Rutter, 1977; Ritvo et al., 1989; see also Hallmayer et al., 2011). In addition to strong genetic influence on the development of autism itself, milder versions of the social, communication, and other difficulties experienced by individuals with ASD have also been documented in unaffected first-degree relatives (i.e., siblings, parents) of those with ASDs (Landa et al., 1991; Bolton et al., 1994; Hughes et al., 1997; Piven and Palmer, 1997; Piven et al., 1997; Folstein et al., 1999; Murphy et al., 2000; Pickles et al., 2000; Bishop et al., 2004; Adolphs et al., 2008; Smith et al., 2009). These results provide evidence that the complex genetic mechanisms that contribute to the development of autism also impact upon other members of families affected by autism. This, then, creates an opportunity to explore the effects of familial/genetic risk factors on various brain and behavioral mechanisms early in life in ASD, through the study of infant siblings of children already diagnosed with ASDs (Rogers, 2009; Yirmiya and Charman, 2010).

Extensive research has been conducted on motor behaviors and motor-related skills in infants who are at high risk for developing autism, with solid implications for our understanding of motor development associated with autism (Iverson and Wozniak, 2007; Rogers, 2009). In a comprehensive review of the autism high-risk infant literature, Rogers (2009) concludes that delays in motor development have been a consistent finding in this population. Of particular note is her conclusion that some important, albeit subtle, repetitive movements, and unusual sensory behaviors appear to emerge earlier in development than impairments in social and communication skills in this population (Rogers, 2009). In this section of the review, we focus on the key findings of the autism early motor development literature, with an emphasis on those motor and motor-related behaviors that are believed to be most relevant to successful communication and language development.

One of the earliest developing motor-related behaviors having associations with language development is the vocal-motor and facial-motor coordination that emerges during face-to-face interactions in the first half of the first year of life (Iverson and Fagan, 2004). During this time, infants begin to engage in coordinated vocal and facial motor activity routines (such as reciprocal vocalizations, imitation of mouth opening, positive/negative facial expressions, and gaze) on a second-by-second timing scale, with both familiar and unfamiliar communicative partners. This motor synchrony reflects interpersonal coordination of listening to and producing vocal-motor activity, which can be considered developmental precursors to the timing pragmatics of interpersonal interaction during conversation (Colonnesi et al., 2012). Existing evidence suggests that the nature and degree of this early infant coordination and tuning of motor activity with others predicts later infant social-emotional and cognitive development in typically developing infants (Feldman et al., 1996).

Yirmiya et al. (2006) measured communicative synchrony in 4-month-old infant siblings of children diagnosed with autism and low-risk infants without a family history of autism during mother-infant interactions. They uncovered evidence for weaker synchrony for infant-led interactions in the high-risk group (see also Brisson et al., 2011). Furthermore, the authors reported that these infants at risk for autism displayed fewer non-verbal requesting behaviors (such as pointing), and performed worse than low-risk infants on the language scales of the Bayley Scales of Infant Development, in follow-up at 14 months of age (Yirmiya et al., 2006). These findings support the hypothesis that risk for autism is associated with impaired vocal-motor coordination synchrony at 4-months of age, and that this has relevance to the later development of linguistic and pre-linguistic behaviors.

Another major stage of links between motor activity and language development occurs during the second half of the first year of life (Bates et al., 1999; Bates and Dick, 2002). Studies have shown that sharp increases in coordinated and repetitive arm movement and hand banging co-occur with the onset of reduplicative babble (i.e., canonical babble; e.g., “baba”) between 6- and 11-months of age in typically developing infants, likely reflecting entrainment of the vocal and manual motor systems (Locke et al., 1995; Iverson et al., 2007; see also Petitto and Marentette, 1991; Petitto et al., 2004). This relationship is robust across typical infants of widely varying age of reduplicative babble/hand banging onset (Eilers et al., 1993; Iverson et al., 2007), as well as children with delayed language, including those with Down Syndrome and those with Williams Syndrome (Cobo-Lewis et al., 1996; Masataka, 2001). Finally, delayed onset of reduplicative babble has been found to be a marker for delays in speech and language in the general population of infants (Oller et al., 1998).

In 2007, Iverson and Wozniak examined the rate of rhythmic arm movements during pre-babble and babble onset sessions in high-risk and low-risk infants. Rates of rhythmic arm movements increased from the pre-babble sessions to the babble-onset sessions in both high-risk and low-risk infants; however, this increase was lower in the high-risk group (Iverson and Wozniak, 2007). In addition, the high-risk infants exhibited delays in reduplicative babble onset and first word use between 5 and 14 months of age, as well as delays in language development at 18 months of age (Iverson and Wozniak, 2007). A related study by Gernsbacher et al. (2008) found that scores on oral-motor (e.g., blowing bubbles) and manual-motor skills (e.g., pointing to request) during home videos distinguished infants who later developed autism from those who were typically developing, as well as infants who were later minimally and highly fluent. Together, these findings suggest that oral-motor and manual-motor skills may contribute to both social-communication and speech/language skills deficits in this population.

Another major stage of links between motor, speech, and language development occurs from approximately 10- to 20-months of age. There is evidence to suggest that typically developing infants learn to understand word-object relationships through repeated episodes of shared joint visual attention to an object (e.g., following a point to look at the ball together) paired with adults verbally labeling the objects (e.g., “ball”) during this period (Baldwin, 1995). This represents a complexity of emerging skills in following and comprehending the motor actions of others in relation to increasingly specific distal targets, and in increasingly dynamic activities and contexts (e.g., Tomasello and Farrar, 1986; Baldwin et al., 1996; Flom et al., 2004).

There is extensive evidence that both young children diagnosed with autism and young toddlers at risk for autism exhibit pervasive impairments in joint attention behaviors. In 2005, Goldberg and colleagues identified deficits in social-communicative behaviors, including responding to joint attention bids, in both 17-month-old high-risk infants and 2-year-old children already diagnosed with autism, compared with typically developing infants and children (Goldberg et al., 2005). In another study, involving 20-month olds diagnosed with autism, Charman (2003) found that declarative, triadic gaze switching was correlated with both language ability and autism symptom severity outcomes at 42 months of age (see also Yoder et al., 2009). Together, these results provide evidence to support the hypothesis that early deficits in the understanding of the gestures and actions of others are present from early in life in this population, and that these deficits are predictive of later social-communication and language deficits in children with autism (see also Rogers, 2009). Given the evidence from typical development, it will also be important to examine potential relationships between early exploratory and locomotor activity and later joint attention and language skills in infants at high risk for autism (see e.g., Campos et al., 2002).

Alongside the development of these social coordination and social-communication aspects of action perception and understanding, there is extensive evidence for more direct, *in vivo* links between gesture and language development in infants and children. Specifically, once infants have mastered the basic understanding of the gestures and actions of other people, they begin to regularly produce and employ increasingly complex communicative and symbolic gestures of their own, furthering their own communications and their language development (Bates and Dick, 2002). For example, the onset of recognitory gesture production, such as putting a cup to one's mouth and pretending to drink, correlates with the onset of vocal naming, both within and across infants between 11- and 16-months of age (Volterra et al., 1979; Shore et al., 1990). Between 18- and 20-months of age, gestures with one meaning are used in combination with words with other meanings, in order for the child to begin to be able to produce longer communications (e.g., point to chair and say “mom” to request that mom sits down; see Bates and Dick, 2002, for discussion). Impairments in the production of recognitory gestures as well as the coordination of speech and gesture during communication are core diagnostic measures of early childhood autism, which are included in the Autism Diagnostic Observation Schedule and the Autism Diagnostic Interview (Lord et al., 1994, 2000).

**Figure 1:**
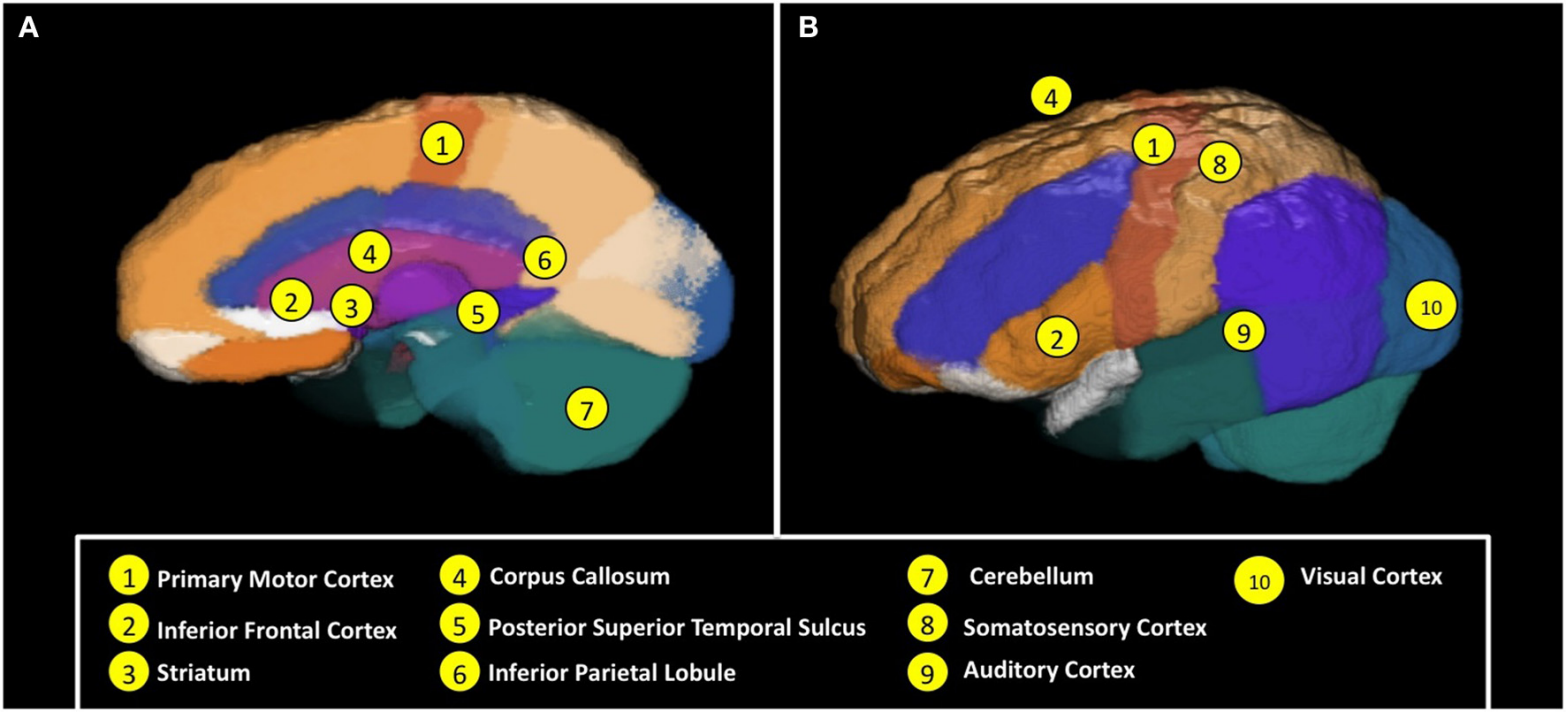
**Neural regions and mechanisms involved in **(A)** motor functioning and action perception, and **(B)** neural coordination and connectivity for sensorimotor and speech/language functioning.** See Table 1 for brief descriptions of these regions and associated mechanisms. Images of an average of 6-year-old child brain generated via the Magnetic Resonance Image database of Sanchez et al. (2012).

In this section, we have reviewed evidence that suggests that infants and young children with autism exhibit deficits and/or delays in a number of motor-related milestones that are believed to reflect critical stages in speech/language and communication development. Indeed, several of these deficits and delays have been found to be concurrently and/or predictively associated with important speech/language and social communication abilities in these infants and children. These motor and motor coordination milestones are likely to be supported by the core motor system and its mediators, including the primary motor cortex, cerebellum, motor-related frontal-striatal connections, visual regions involved in action perception, and a distributed system for sensorimotor integration (see Figure 1 and Table 1 for more information). These findings have clear implications for how motor-related interventions might be used to facilitate and support speech/language and communication development in this population, which is the focus of this review. Before we address this, however, we discuss the evidence for deficits in the motor resonance (i.e., “mirror neuron”) system in individuals with autism. This system, which is involved in “mirroring” the actions of others within our own motor planning (i.e., premotor cortex) system, has been proposed to impact upon language development directly (Oberman et al., 2005), or to index social engagement with relevance for speech/language and social communication development in ASD.

**Table 1:** **Brain regions and mechanisms associated with motor aspects of language development**.

**Number (see Figure** 1)	**Brain region or mechanism**	**Description**
1	Primary motor cortex	Primary cortical generator of motor activity, both simple and complex.
2	Inferior frontal cortex	Motor planning region, and key region of the frontal mirror neuron system; also includes Broca's area. Includes representations of hand and mouth actions, and has been implicated in links between hand and mouth actions that facilitate speech/language production and development.
3	Striatum	Portion of the subcortical basal ganglia system, involved in the modulation of movement; affected by inputs from motivational systems.
4	Corpus callosum	Bundle of neural fibers that connect the left and right hemispheres of the brain, facilitating inter-hemispheric communication and coordination.
5	Posterior superior temporal sulcus	Cortical region involved in biological motion perception. Key region of the posterior mirror neuron system, which has been specifically implicated in perceptual aspects of action encoding and understanding.
6	Inferior parietal lobule	Cortical region involved in the association and integration of sensory information. Key portion of the posterior mirror neuron system, which has been specifically implicated in goal-related aspects of action understanding.
7	Cerebellum	Neural region involved in the coordination, precision, and timing of movement, motor learning, and motor integration.
4, 8, 9, 10	Neural integration and connectivity	Both motor and language functioning require coordination and integration across multiple sensory modalities and hemispheres. For example, motor planning and motor coordination require integration of information from visual and motor cortices. Similarly, speech perception requires visual-motor/auditory integration (e.g., mouth movement, speech sounds), and meaningful/iconic language involves the integration of multiple real-world experiences with objects that are encoded within and across the visual, somatosensory, motor, and auditory cortices.

## Motor resonance deficits in individuals with autism

Extensive research, particularly over the past 15 years, has provided convincing evidence that our motor system “resonates” the actions of others that we view, hear, or view and hear (di Pellegrino et al., 1992; Rizzolatti et al., 1996; Iacoboni et al., 1999; Kohler et al., 2002; Gazzola et al., 2006). That is, our motor planning and related action production systems in pre-motor and other regions of the cortex appear to “mirror” the actions of observed others onto our own action/motor planning system (e.g., Inferior Frontal Gyrus, Inferior Parietal Lobule, Superior Temporal Sulcus; see Figure 1 and Table 1), presumably allowing us to better represent and understand the nature and details of the actions and activities of others (Rizzolatti and Craighero, 2004). This “mirror neuron” system (MNS) has been proposed to underlie a number of critical social-interactive and social-communicative skills, including imitation, language development, empathy, and understanding the social perspectives and intentions of others (Iacoboni and Dapretto, 2006). Following an initial suggestion that impairments in mirror neuron functioning may play an important role in the behavioral deficits observed in individuals with autism in 2001 (Williams et al., 2001), behavioral and neuroimaging research has sought to test this hypothesis. Although the findings are somewhat mixed, and there is particular debate about behavioral data on MNS functioning and its proposed relationship to imitation functioning in the literature (Southgate and Hamilton, 2008; see also Hamilton, 2009), the hypothesis of impaired motor resonance in individuals with ASD has generally been supported in the experimental behavioral and brain imaging literatures (Oberman and Ramachandran, 2007; Becchio and Castiello, 2012; Enticott et al., 2012; Oberman et al., 2012).

Despite extensive evidence for reduced visuomotor resonance in individuals with autism, it is clear that the MNS is not entirely “broken” in this population. For example, individuals with ASD have been found to exhibit normal motor interference during simultaneous execution-observation of meaningless arm movements (e.g., Gowen et al., 2008; see Becchio and Castiello, 2012, for review). Most relevant to the current review, Oberman et al. (2008) used electroencephalography (EEG) mu suppression to uncover evidence for normal MNS activation during the observation of the actions of familiar people, but reduced MNS activation during the observation of the actions of unfamiliar people, in children with autism. These data provide direct evidence that the MNS of children with autism is, in fact, capable of responding normally to the actions of others. Along these same lines, a study by Pierce and Redcay (2008) used functional Magnetic Resonance Imaging (fMRI) to uncover evidence that the Fusiform Face Area (FFA) is also activated normally in response to familiar faces, but not in response to unfamiliar faces, in children with autism.

Like the MNS, evidence had generally supported the hypothesis of impaired FFA functioning in individuals with autism prior to this. Together, these findings on familiarity effects in social processing (i.e., MNS, FFA) are consistent with the hypothesis that lack of social and/or emotional familiarity with, or interest in, unfamiliar others may be driving reduced activation of social brain networks, including the MNS, in children with autism. One distinct possibility is that children with autism exhibit reduced social interest and/or social-cognitive attention for strangers, relative to other children. This hypothesis receives support from event-related potentials (ERPs) EEG evidence that very young children with autism exhibit reduced late frontal cortex activity in response to unfamiliar faces (Dawson et al., 2002). More specifically, Dawson et al. (2002) found that both typically developing children and children with developmental delays without autism showed larger amplitude ERPs in response to unfamiliar relative to familiar faces, suggesting increased neural activity for the processing of unfamiliar people. However, children with autism did not exhibit this “interest in strangers” effect. In the same study, all three groups of children did exhibit differential brain responses to familiar versus unfamiliar toys, suggesting that this difference in children with autism reflected a lack of neural activity and cognitive processing specifically for unfamiliar people (see also Oberman et al., 2008; Pierce and Redcay, 2008; Becchio and Castiello, 2012; Dawson et al., 2012).

In summary, evidence suggests that individuals with autism exhibit reduced or absent motor resonance activity during the observation of the actions of unfamiliar others. While it was initially suggested that this reduced/absent activity reflects a “broken” MNS (Williams et al., 2001; Oberman and Ramachandran, 2007), more recent results and analysis suggests that reduced/absent mirror neuron activity may reflect reduced social engagement in this population (Oberman et al., 2008; Becchio and Castiello, 2012). Taking the latter view, in the current review, we consider early behavioral interventions that teach speech/language and social communication skills in the specific context of socially engaging synchronous motor activities as a potential motor-related pathway to increasing social-communication and language skills in this population.

## Interventions

Delays and impairments in motor and motor-related development in infants and children with autism have implications for early intervention in this population. Whereas previous reviews have focused on interventions aimed at improving sensory and motor functioning (Baranek et al., 2008) and other ASD-related behaviors (Sowa and Meulenbroek, 2012), here we review and discuss existing and emerging motor interventions that are more directly relevant for increasing social-communication and language skills in toddlers and children with autism. We focus particular attention on their theoretical and practical relationships to motor theories of social-communication and language development, as well as to their existing evidence base. In examining the evidence base, we consider several types, or levels, of evidence (see Table 2). These include case study reports, which can involve descriptions of multiple children but without experimental controls. Next, we consider experimental single subject designs, which exert experimental control through the use of baseline recordings of varying lengths across multiple children, thus more reliably attributing intervention effects to intervention onset. Along with these, we include small-scale pseudo-experimental research designs, whereby children are assessed pre- and post-intervention, but without a comparison control group to account for potential naturally occurring developmental improvements in the target behaviors. Finally, we consider large-scale experimental group studies, Randomized Controlled Trials (RCTs; efficacy trials), and RCTs conducted in community settings (effectiveness trials). As ASDs are a unique class of developmental disorders, we focus our review specifically on the evidence-base for the efficacy and effectiveness of each intervention for children with ASDs. Finally, we focus exclusively on interventions for non-verbal and minimally verbal children, because there are existing evidence-based interventions that are effective for more verbally able children with autism (Koegel, 2000). We start with sign language intervention, which has previously been proposed to be a mechanism for linking motor-based gesture and speech and language development in these children.

**Table 2:** **Levels of evidence for each intervention**.

**Intervention**	**Brief description**	**Case reports**	**Experimental single-subject designs (multiple baseline designs, reversal designs) and small-scale pseudo-experimental group designs**	**Large experimental study**	**Randomized controlled trial**	**Community-based randomized controlled trial**	**Summary of evidence**
**AUGMENTATIVE AND ALTERNATIVE COMMUNICATION (AAC) INTERVENTIONS**							
Sign language (SLT)	Teaches child to use hand, arm, facial, and other actions to create symbolic communications	5+	15+	1	0	0	Extensive research base. Weak but mixed evidence for learning of sign language. Weak evidence for learning of speech. Weak evidence for learning of speech via sign plus speech training. See Schwartz and Nye (2006).
		1. Fulwiler and Fouts (1976)	1. Carr et al. (1987)	1. Layton (1988)			
		2. Brady and Smouse (1978)	2. Barrera and Sulzer-Azaroff (1983)	2. Yoder and Layton (1988)			
			3. Sundberg et al. (2000)				
Picture exchange communication system (PECS)	Teaches child to exchange pictures with others, in order to make requests and comment	5+	5+	1	3+	1	Extensive research base. Moderate evidence for both picture-based and verbal communication gains. See Sulzer-Azaroff et al. (2009).
		Speech:	Speech:	Communication:	Speech:	Speech:	
		1. Webb (2000)	1. Charlop-Christy et al. (2002)	1. Lerna et al. (2009)	1. Yoder and Stone (2006a)	1. Gordon et al. (2011)	
		2. Bondy and Frost (1994)	2. Carr and Felce (2007a)		Communication:	Communication:	
		Communication:	Communication:		2. Yoder and Lieberman (2010)	2. Howlin et al. (2007)	
		3. Anderson et al. (2007)	3. Travis and Geiger (2010)				
		4. Malandraki and Okalidou (2007)	4. Greenberg et al. (2012)				
**MOTOR-BASED BEHAVIORAL INTERVENTIONS**							
Prompts for restructuring oral muscular phonetic targets (PROMPT)	Uses physical prompts to the vocal apparatus, as well as social, kinesthetic, and proprioceptive awareness, to increase speech and language.	0	1	0	0	0	Limited evidence in ASD.
			1. Rogers et al. (2006)				
Auditory motor mapping treatment	Teaches the pairing of sounds with motor actions during picture-based word teaching in order to facilitate vocalization	0	1	0	0	0	Limited evidence in ASD.
			1. Wan et al. (2011)				
**ELECTROMAGNETIC BRAIN STIMULATION INTERVENTIONS**							
Transcranial direct current stimulation (TdCS)/Transcranial magnetic stimulation (TMS)	Electromagnetic brain stimulation procedures.	0	1	0	0	0	Limited evidence in ASD.
			1. Schneider and Hopp (2011)				
**INTERVENTIONS TARGETING SYNCHRONOUS MOTOR ACTIVITIES**							
Early start denver model (ESDM)	Integrative model of play-based behaviorist/operant teaching methods within a comprehensive developmental framework.	5	20+ (includes PRT evidence)	1	1+	1	Extensive research base (includes PRT evidence). Moderate evidence for verbal and non-verbal communication gains. See Warren et al. (2011).
		1. Voos et al. (2012)	1. Vismara et al. (2009)	1. Baker-Ericzén et al. (2007)	1. Dawson et al. (2010)	1. Rogers et al. (2012)	
		2. Vismara and Rogers (2008)	2. Vismara and Lyons (2007)				
			3. Pierce and Schreibman (1995)				
			4. Stahmer (1995)				
Reciprocal imitation training (RIT)	Uses reciprocal imitation and behaviorist principles to teach the child to imitate the motor actions and gestures of others in a play context.	0	3+	1	0	0	Moderate evidence for non-verbal communication and imitation gains.
			1. Ingersoll and Schreibman (2006)	1. Ingersoll (2010)			
			2. Cardon and Wilcox (2011)				

### Augmentative and alternative communication (AAC) interventions

#### Sign language training

**Figure 2:**
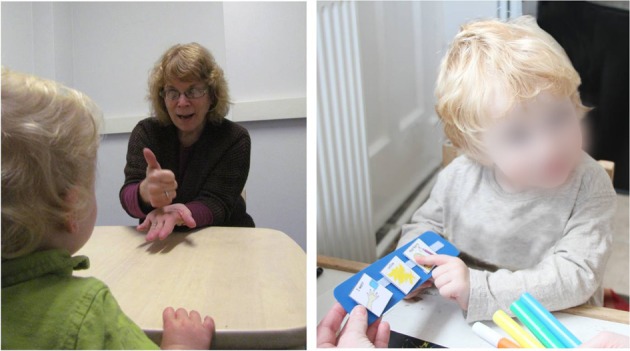
**Augmentative and Alternative Communication (AAC) interventions.** Child and therapist engaged in Sign Language Training (left) vs. Picture Exchange Communication System (PECS) training (right). Sign Language Training (SLT) uses behaviorist imitation and prompting methods to teach children to use hand, arm, facial, and other body actions to produce symbolic communications. The Picture Exchange Communication System (PECS) uses behaviorist methods to teach children to hand one or more pictures to a variety of communicative partners, in order to request items/activities, respond to simple questions, and comment.

For non-verbal autistic children, training in augmentative and alternative communication (AAC) offers a route via which these individuals can begin to communicate. The two most widely accepted AAC strategies are Sign Language Training (SLT; Carr et al., 1978) and the Picture Exchange Communication System (PECS; Bondy and Frost, 1994; Frost and Bondy, 2002; see Figure 2). Research suggests that educators believe that both of these strategies are viable options for teaching communication skills to children with autism displaying severe deficits in communication skills (Stahmer et al., 2005).

Given the strong links between gesture and verbal communication in typically developing infants, including those described in the sections above, the use of SLT to facilitate speech in developmentally delayed populations has a logical theoretical basis. Indeed, early studies investigating the impact of SLT on children with autism yielded promising results, in both the communicative and social domains (Miller and Miller, 1973; Bonvillian and Nelson, 1976; Fulwiler and Fouts, 1976; Brady and Smouse, 1978; Konstantareas, 1984). Contrary to expectations, however, these marked improvements in communication did not include speech development. Furthermore, the effectiveness of sign language alone as a means to facilitate speech in non-vocal autistic children was quickly called into doubt; as was the degree of experimental control employed by early research in this area (Carr et al., 1978; Carr, 1979; see Table 2).

Following the recognition that SLT did not lead to meaningful increases in speech in children with autism, studies utilizing training sessions that focused on coupling sign language with other forms of training (e.g., speech intervention plus SLT) were conducted. This combined intervention approach proved to be more effective than sign language alone for eliciting spoken vocabulary in nominally verbal autistic children (Brady and Smouse, 1978; Layton and Baker, 1981; Konstantareas, 1984; Yoder and Layton, 1988). However, when considering this research, it is important to note that the participants in these studies had existing verbal skills. Therefore, it has yet to be examined whether SLT in any form can elicit verbal communication gains in non-vocal autistic children. Moreover, outcomes following SLT are extremely and unusually variable. For example, although a small number of individuals with autism adopt sign language as their primary mode of communication and appear to readily learn signs (Barrera et al., 1980; Stull et al., 1980), others are unable to attain even the most basic signing skills (Webster et al., 1973; Brady and Smouse, 1978; Carr et al., 1978).

Despite decades of research into SLT as an effective tool for teaching those with ASD, the evidence that it leads to novel and/or increased functional uses of communication, speech, and language in this population is weak. Those who suggest that sign language, or total communication (sign plus speech), may serve to increase such skills in autistic individuals often base their arguments on single-subject research (Carr et al., 1978, 1987; Casey, 1978; Cohen, 1979; Schepis et al., 1982). Although rich in detail, the majority of these more promising SLT studies provide no measure of fidelity of implementation, few explored generalizability, and many fail to disclose sufficient detail for either clinical application or experimental replication (Millar et al., 2000; Schwartz and Nye, 2006). In their review of SLT in this population, Layton and Watson (1995) maintain that, despite extensive training, the majority of nonverbal children fail to develop any form of vocalization and, at most, learn a few basic signs, as a result of SLT. In a more recent review of sign language and communication gains in children with autism, Schwartz and Nye (2006) conclude that teaching communication through signing does not serve as an effective intervention to improve either sign or oral language communication in children on the autism spectrum (see also Millar et al., 2000).

While the poor results of SLT have often been overlooked in the literature, some attempt has been made to explain these findings. One proposed explanation for the relative failure of SLT is that the successful acquisition and use of sign language as a communicative tool is dependent on the ability to form a variety of manual-motor signs and there are many individuals with ASD who do not possess the fine motor skills required (Bonvillian and Blackburn, 1991; Seal and Bonvillian, 1997; National Research Council, 2001). Similarly, Mirenda and Erickson (2000) outline “the three I's” that contribute to successful sign language acquisition: *imitation, iconicity*, and *intelligibility*. They maintain that children with autism demonstrate a lack of imitation, symbolic representation, and motor coordination/planning skills, while the successful acquisition and use of sign language relies largely on the possession of these abilities (see Table 1 and Figure 1 for relevant neural mechanisms). In each of these proposed explanations, deficits and delays in motor and motor-related skills are key to explaining why children with autism generally fail to develop both sign language-based communication and speech and language skills as a result of SLT.

#### Picture exchange communication system (PECS)

Given the lack of meaningful progress as a result of SLT, it is unsurprising that the field has turned its attention to other AAC training practices. The PECS is a form of AAC that utilizes pictures as its primary medium of communication and, like SLT, has foundations in behaviorist principles. The primary goal of PECS is to establish and increase spontaneous communication within social contexts, which is initiated through picture-based communication (Bondy and Frost, 1998). PECS is a structured and manualized intervention program that is designed to teach children to communicate via a book containing detachable pictures (see Figure 2).

The PECS protocol is divided into six phases, each designed to expand upon the child's development during the previous phase. In Phase I, the child is taught to hand a single picture to another person, in exchange for a desired item or activity (e.g., a ball). In Phase II, the child is taught to exchange pictures with multiple people in multiple environments. Phase III teaches the child to discriminate and select among pictures for a number of desired items. Phase IV teaches the child to produce simple sentence structures (e.g., “I want ____.”) using pictures, which are then handed to communicative partners using a sentence strip (see Figure 2). Finally, Phases V and VI teach responding to simple questions and commenting, using pictures. The child typically progresses from basic picture-based requesting, to more advanced picture-based responding and spontaneous commenting (Bondy and Frost, 1998). The surface appeal of PECS over sign language is understandable given that it does not rely on the communicator possessing complex fine motor skills, nor does it burden the communicator with learning a completely new language (Bondy and Frost, 1994). Furthermore, the gains facilitated by PECS do not appear dependent upon the child possessing pre-existing skills (Bondy and Frost, 2002; Yoder and Stone, 2006a,b), and PECS appears to be readily learned by children with autism as well as other developmental disorders (Schwartz et al., 1998; Mirenda and Erickson, 2000; Charlop-Christy et al., 2002; Ganz and Simpson, 2004; Preston and Carter, 2009).

Although not initially developed to teach spoken language, a large and growing body of evidence demonstrates that PECS can assist with spoken language development in children with autism with existing, albeit limited, verbal skills (Bondy and Frost, 1994; Liddle, 2001; Charlop-Christy et al., 2002; Kravits et al., 2002; Magiati and Howlin, 2003; Anderson et al., 2007; Carr and Felce, 2007a; Carré et al., 2009; Jurgens et al., 2009; Preston and Carter, 2009; Sulzer-Azaroff et al., 2009; Greenberg et al., 2012). Early non-experimental, retrospective research by Bondy and Frost (1994) suggested that after one year of PECS usage, 76 percent of 66 young children developed speech either as their sole means of communication or alongside picture communication. Following a series of experimental single-subject design studies suggesting positive effects on both communication and speech as a result of PECS intervention, several large scale experimental studies have provided further strong and convincing evidence that PECS increases both social-communication and speech/language skills in children with autism. Indeed, increases in spoken and socio-communication skills through PECS training appear to be as prominent as in speech-based interventions (Yoder and Stone, 2006a,b; Lerna et al., 2012). For example, Yoder and Stone (2006a) compared the effects of PECS and Responsive Education and Prelinguistic Milieu Teaching (RPMT) in 36 toddlers and young children with autism. Both interventions were implemented for the same length of time, and at the same intensity. After six months of training, it was found that PECS training resulted in increased verbalizations, both in terms of frequency and range of words. Although children in both treatment groups were found to have made similar speech-related improvements by their six-month follow-up, the authors highlight that these results provide evidence that PECS leads to more swift speech development when compared to RPMT. Similarly, recent research by Lerna et al. (2012) compared the efficacy of PECS with Conventional Language Therapy (CLT) in a group of preschool children with ASD. Following six months of treatment, those receiving PECS demonstrated significant improvements in their joint attention, requesting, and imitation skills.

Although RCT's are severely lacking in the field of autism education research (Carter and Wheldall, 2008; Preston and Carter, 2009), the few large-scale examinations that have involved such advantageous designs have also replicated the promising data on PECS (Table 2). For example, a recent school-based RCT of PECS versus Treatment As Usual (TAU) by Howlin and colleagues highlighted gains in spontaneous requesting through picture use, speech, or both (Howlin et al., 2007). Gordon and colleagues (2011) examined these same data from 84 autistic children across 15 British schools, observing changes in spontaneous communication following immediate, delayed, or no PECS training. They found that children who had received immediate treatment demonstrated significant increases in both spontaneous speech/vocalizations, and in their usage of PECS. Furthermore, Carr and Felce (2007b) compared a PECS training group (*n* = 24) with a no treatment control group (*n* = 17), and uncovered evidence for significant increases in linguistic communicative initiations that included the use of spoken words within the PECS treatment group, and no improvements in such skills within the no-treatment control group. This, again, demonstrates the efficacy of PECS in eliciting both verbal and non-verbal communicative behaviors in children with autism.

It is worth noting that children with autism typically exhibit increases in speech during Phases IV and V of PECS training (Charlop-Christy et al., 2002; Ganz and Simpson, 2004). During these Phases, they are learning to use a larger number of pictures, and have also started to point rhythmically to sentences, often syllable by syllable (Frost and Bondy, 2002). Prior to Phase V, children are taught to (a) communicate with pictures (Phase I), (b) travel and seek their communication partner (Phase II), (c) discriminate individual pictures and what they each represent (Phase III), and (d) structure sentences through the use of a string of picture cards (Phases IV and V; Frost and Bondy, 2002). Phase IV is also the period during which a time delay procedure is used by the therapist, whereby she or he pauses after speaking the first portion of the picture-phrase (e.g., says “I want … ”) and waits 3–5 s for the non-verbal or minimally verbal child to verbalize the label for the item they have requested (e.g., “ball”) before providing the item to the child. In this instance, the child's rhythmic pointing to the pictures (e.g., I-want-BALL) continues as the therapist stops speaking, potentially facilitating the child's verbalization of the target item (e.g., “ball”). As mentioned, a plethora of research has demonstrated the link between the onset of speech, and the development of coordinated hand banging gestures. It is possible that the speech gains observed in many children during this phase of PECS are a reflection of this link, with implications for the potential importance and validation of hand-mouth motor plans, as described in relation to auditory motor mapping intervention below.

In sum, although there are strong links between motor-based symbolic gesture and speech development in typical infants and children, extensive research suggests that there is no robust link between SLT and increased speech in children diagnosed with autism. Although many children with autism do not readily learn the use of signs, a large body of evidence demonstrates the ease with which they acquire picture-based communication via PECS, suggesting that it is not an inability to learn that is attributable to their difficulties in sign language learning in this population. Furthermore, as outlined, research also suggests stronger links between speech development and PECS training vs. SLT, in children with autism. Some have proposed that difficulties in sign language learning are due to impairments in fine motor skills (Bonvillian and Blackburn, 1991; Seal and Bonvillian, 1997; National Research Council, 2001), whereas others have argued that it is a combination of *imitation skills, iconicity*, and *intelligibility* that present challenges to this population (Mirenda and Erickson, 2000). Next, we examine several more directly motor-based interventions that are currently under development to address social-communication and speech/language skills for this population.

### Motor-based behavioral interventions

While sign language is a gesture and motor-based intervention, there are other behavioral interventions that take an even more direct approach to addressing motor aspects of speech production. These include interventions that involve direct manipulations of the mouth and other sound-producing structures, and those that make more direct low-level links between hand and oral motor activity. Here, we describe research on the two interventions of this type that have been studied in relation to children with autism.

#### Prompts for restructuring oral muscular phonetic targets (PROMPT)

**Figure 3:**
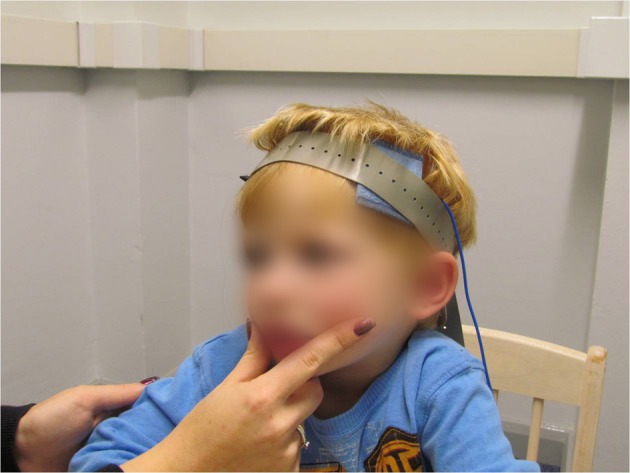
**Motor-based behavioral intervention and electromagnetic brain stimulation intervention.** Child and therapist engaged in Prompts for Restructuring Oral Muscular Phonetic Targets (PROMPT) intervention. The child is also wearing a Transcranial Direct Current Stimulation (tDCS) electrode band. These two techniques are typically implemented separately. Here, the PROMPT therapist administers a physical prompt to the child's vocal-motor system, in order to facilitate production of a speech target, while the tDCS electrode applies a direct current to the left inferior frontal cortex.

One intervention targeting the neuromotor underpinnings of speech production is the Prompts for Restructuring Oral Muscular Phonetic Targets (PROMPT; Chumpelik, 1984) model. PROMPT goes beyond auditory and visual input, integrating neuromotor principles with social, kinesthetic, and proprioceptive awareness to facilitate the production of clear sounds, speech, and language (Hayden, 2002). In addition to manipulating sound-producing structures, PROMPT places importance on body movement and stability. A typical PROMPT session involves play-based or naturally occurring activities that are likely to encourage interaction initiations from the child. Using these initiations or motivators as a therapeutic opportunity, the clinician then uses vocal modeling and physical manipulations of the child's speech mechanisms as they attempt verbalization. Such manipulations include touch, pressure, positioning, and movement to promote structural integration within the child's vocal apparatus (Hayden and Square, 1994; see Figure 3).

A PROMPT is available for every vowel or consonant in the English language, as well as for every single or combined speech-sound utterance. Specifically, therapists may use *parameter* prompts to provide support to the jaw and facial muscles; *surface* prompts to aid the formation of speech sounds and their associated timings and transitions; *syllable* prompts to teach the critical combination of jaw support and lip positioning required to produce legible syllables; and finally, *complex* prompts may be administered when teaching the formation of single sounds (Hayden, 2006) Due to these multiple types of prompts, the PROMPT model can be used to build upon the motor skills of children at all stages of speech production, from first-word attempts to the production of more intelligible speech. Throughout the course of intervention, manual prompts are gradually faded as the child demonstrates heightened oral awareness and control.

The PROMPT intervention method has been examined in a number of studies, although most report on individual case studies. For example, Square et al. (2000) examined six young children with language and phonological disabilities and, following PROMPT intervention, discovered increased accuracy of target word production, and generalization of abilities to untrained words. Gains were also noted in overall communication, social interaction, and intelligibility. Furthermore, Square et al. (1986) noted the efficacy of PROMPT training in three patients with acquired apraxia, whilst a recent study by Ward et al. (2009a,b) found gains in intelligibility, consonant accuracy, and generalized vocal improvements in children with cerebral palsy and speech impairments. In a case study of a severely apractic-aphasic male, PROMPT training for 41 weeks was associated with maintained articulation accuracy in a set of core functional words and phrases (Freed et al., 1997). Finally, although Dodd and Bradford (2000) found no effect of PROMPT intervention in three boys with phonological impairment without articulation disorders, Grigos et al. (2010) discovered increased articulation accuracy in a single subject with severe articulation impairment.

To date, only one published study has explored the effects of the PROMPT method in children with ASD. Rogers et al. (2006) randomly assigned 10 non-verbal children with autism to receive one of two interventions: the Denver Model (a play-based program based on reciprocal communication and social engagement; Rogers et al., 2000), or PROMPT. All participants received 12 weeks of treatment and were assessed for their use of novel words and phrases throughout the intervention, as well as for the maintenance of such functional communication at three weeks post-treatment. Assessments throughout and following intervention revealed that 80% of participants exhibited increases in spontaneous, functional words. In light of the small sample sizes, and in the absence of group comparisons, this study can only be considered a series of non-experimental case studies. Nevertheless, these preliminary findings do suggest potential promise for the use of the PROMPT model with autistic children, and future research should endeavor to examine a larger sample of autistic children in a RCT or other experimental assessment of the PROMPT intervention.

#### Auditory motor mapping training

Auditory-Motor Mapping Training (AMMT; Wan et al., 2009) is a recently developed multi-component intervention targeting the development of speech output through singing, motor activity, and imitation (Wan et al., 2010a). Based upon the hypothesis that individuals with autism have a deficient MNS, AMMT was designed to train sound-articulation associations by engaging multiple neural networks (Wan et al., 2010b). In essence, the goal of AMMT is to teach the pairing of sounds with motor actions in order to facilitate vocalizations.

During a typical AMMT session, a target word or phrase is introduced, and the therapist repeatedly intones the word or phrase while simultaneously tapping a pair of drums tuned to different pitches. The child is then encouraged or gently guided to imitate these actions, while being presented with images of the target object, action, or person. These three components are believed to work together to promote increased interactions between the auditory and motor systems, strengthening the likelihood of intelligible and functional speech production. For example, the use of intonation as opposed to simply speaking is designed to heighten bilateral fronto-temporal network activation—an area associated with components of the MNS (Brown et al., 2004; Ozdemir et al., 2006). Similarly, the engaging use of percussion has been implicated in the activation of a sensorimotor network responsible for articulatory and oro-facial movements, as well as stimulating the mapping of sounds to actions through increased bilateral activation in the fronto-parietal motor-related network (Meister et al., 2003, 2009; Lahab et al., 2007). The third component, imitation, is designed to encourage learning, and is argued to alter the responses in the MNS (Catmur et al., 2007).

One small-scale study describing several cases has been reported on AMMT as an intervention for children with autism. Wan et al. (2011) examined 6 non-verbal children with autism who each received five AMMT sessions per week throughout an eight-week period. All children were assessed on their vocal production at baseline, during the therapy, and following completion of treatment. The authors report that word and phrase articulation improved notably in all of the children, with improvements including verbalizations of both trained and untrained words. Although promising, the results from this case study series must be interpreted with caution, particularly in regards to whether or not the intervention was driving the observed effects. To date, there has yet to be an experimental study examining the efficacy of AMMT for treating children with ASD. On the other hand, the results from these initial case studies serve as a promising starting point to initiate larger-scale and experimental studies of AMMT.

### Electromagnetic brain stimulation interventions

#### Transcranial direct current stimulation and transcranial magnetic stimulation

Transcranial Direct Current Stimulation (tDCS) and Transcranial Magnetic Stimulation (TMS) are relatively new methods via which low intensity intracranial electrical current is applied to the cerebral cortex (see Figure 3). The current is the result of a fluctuating magnetic field that comes from external resources, and tDCS and TMS are considered non-invasive brain stimulation procedures (Pascual-Leone and Walsh, 2002; Gandiga et al., 2006). In tDCS, a relatively weaker direct current is applied constantly through electrodes attached to the scalp above a brain region of interest. This current alternates the neuronal excitability in either a positive or a negative manner, leading to changes in brain function (Nitsche et al., 2008). A combination of tDCS and other rehabilitative treatments has been studied in relation to motor training protocols (Hummel and Cohen, 2005). TMS has been successfully used to alleviate, or attempt to alleviate, neurological symptoms associated with stroke (Oliveri et al., 1999), epilepsy (Fregni et al., 2006), and a variety of psychiatric disorders (Lisanby et al., 2002). Most relevant to the current review, repetitive TMS (rTMS) has been shown to improve naming abilities in adults with chronic aphasia resulting from stroke (Martin et al., 2009; see also Mimura et al., 1998; Winhuisen et al., 2007).

In 2011, Schneider and Hopp applied tDCS to the left dorsolateral prefrontal cortex in a group of 10 minimally verbal children with autism, in order to examine the possibility of syntax acquisition as a result of tDCS (Schneider and Hopp, 2011). They found significant improvements in behavioral performance on a basic subject-verb-object sentence sub-test of the Bilingual Aphasia Test. Based on these promising group case study findings, the authors have proposed that additional research should be conducted in this area (see also Sokhadze et al., 2009). Furthermore, the results of a recent small-scale experimental study of adults with Asperger's Syndrome further suggest that the application of rTMS may, indeed, prove useful for improving language skills in those with ASD (Fecteau et al., 2011). It is important to note, however, that there are notable risks associated with both tDCS and TMS, some of which have particular practical, medical, and ethical implications for the application of these technologies to individuals with ASDs (see below for further information).

In sum, there are at least three relatively new strongly motor-related interventions for potentially treating speech and language skills in young non-verbal and minimally verbal children with ASD. Interestingly, each of these interventions has precisely one published paper on their usefulness in treating this population. Also of interest, is that the results of these studies all provide promising results. This being the case, however, none of these studies were experimental in nature and, instead, took the form of a small-scale pseudo-experimental design in each case. It is clear that experimental research is now warranted in order to examine the potential efficacy and effectiveness of these novel interventions. However, the application of one of these interventions, tDCS/TMS, presents some practical, medical, and ethical challenges in relation to children with autism (see Discussion and Future Directions, below, for further information).

### Interventions targeting synchronous motor activities

Play-based intervention methods based upon the application of behavior analytic procedures are well-established and commonly used techniques for teaching children with autism difficulties to engage in new social, communication, play, language, and other behaviors. These interventions utilize operant teaching methods, including behaviorally-defined targets, contingent reinforcement (e.g., access to items and activities, descriptive praise), physical and verbal prompts, and shaping and fading procedures, to target skill development, while allowing the child a great deal of choice in play activities. Extensive and large-scale experimental research studies have shown that these interventions can increase generalized and spontaneous language and communication skills (Koegel and Koegel, 2006), improve social and play skills (Pierce and Schreibman, 1995; Stahmer, 1995), decrease inappropriate behavior (Koegel et al., 2005), and improve academic motivation and performance (Koegel et al., 2010).

More recently, researchers have worked to combine developmental and behavioral intervention approaches, whereby operant teaching methods are utilized to target skills within a strong developmental framework in a play-based context. Most relevant to the current review, two of these developmental-behavioral interventions specifically target social-reciprocity and social engagement in the context of synchronous motor activities, which may represent a potential motor-related pathway to increasing social-communication and language skills in this population.

#### Early start denver model (ESDM)

The Early Start Denver Model (ESDM) is an integration of a particular play-based behavior analytic approach, Pivotal Response Treatment (PRT), with developmental intervention methods designed to increase reciprocal social relationships and social engagement in young children with autism (Rogers and Dawson, 2009a). As with other play-based behavior analytic interventions, ESDM places a major focus on child motivation. Unique to the ESDM, however, is that the course of intervention for each child is based on a structured Curriculum Checklist, specifically targeting developmentally-based social-interactive skills, social communicative skills, cognitive skills, language, imitation, fine and gross motor skills, self-help skills, and adaptive behaviors (Rogers and Dawson, 2009b). The ESDM has an experimental evidence base, including an impressive and extensive set of previous experimental research studies on PRT and a large-scale RCT of the efficacy of the ESDM itself in toddlers on the autism spectrum (Dawson et al., 2010). In this study, 48 toddlers between 18 and 30 months of age were randomly assigning to either the ESDM intervention group, or to a group referred for community-provided intervention. Across the two-year training period, those in the ESDM intervention group demonstrated significant improvements in scores of adaptive behavior and IQ (including Verbal IQ/Language) when compared to both baseline scores and the community-referral group. These toddlers also exhibited more positive changes in the severity of their autism diagnosis. That is, in comparison with community intervention, ESDM intervention led to more children experiencing changes in their diagnosis from Autism to PDD-NOS.

#### Reciprocal imitation training (RIT)

**Figure 4:**
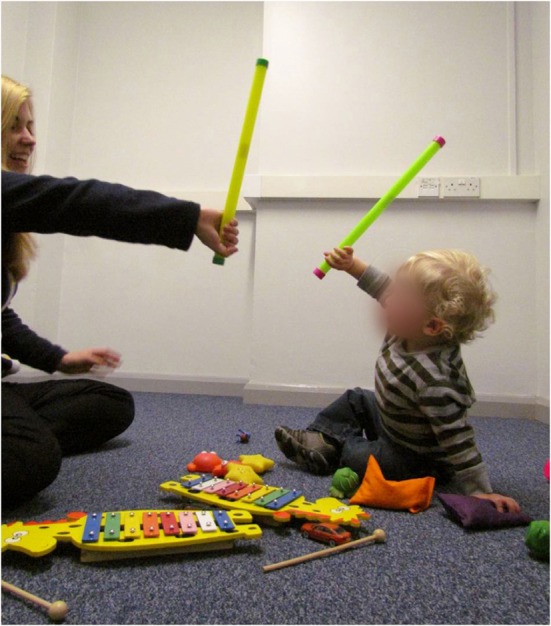
**Interventions targeting synchronous motor activity.** Child and therapist engaged in Reciprocal Imitation Training (RIT). RIT involves the therapist imitating the child's actions and gestures, and also modeling developmentally-appropriate actions and gestures for the child to imitate, in a play context. The child is encouraged and prompted to imitate, until regular spontaneous reciprocal imitation is established.

Reciprocal Imitation Training (RIT) is a recently developed intervention that primarily targets object and gesture-based action imitation in children with autism (Ingersoll and Schreibman, 2006). Following the same basic principles as PRT and the ESDM, RIT is child-directed and incorporates motivational strategies to facilitate engagement and learning. However, RIT was developed on the grounds that naturalistic action imitation is a critical social learning tool that contributes to rapid advances in social and cognitive development in infants and children (Meltzoff and Moore, 1977; Bates et al., 1979; Fiese, 1990; Uzgiris, 1991; Carpenter et al., 1998; Charman et al., 2000, 2003; Stone and Yoder, 2001), and is significantly impaired in children with autism (Curcio, 1978; Dawson and Adams, 1984; Stone et al., 1997; Williams et al., 2004). In essence, the RIT intervention sessions are designed to create ongoing turn-taking situations whereby the therapist and child reciprocate imitation of each other's actions (see Figure 4). The RIT therapist imitates the child's actions with objects, gestures, movements, and vocalizations, and strategically incorporates the modeling of new developmentally-appropriate actions or gestures approximately once every one to two minutes. The child is provided with up to three actions to imitate in a naturalistic play context, before being physically prompted to imitate the fourth action if and when he or she does not engage in any imitation. As the child learns to reciprocate this imitation, and in turns becomes more attentive and socially engaged with the therapist, the need for prompting decreases until child-therapist imitation is a natural part of the play routine. The ultimate goal of RIT is to increase the generalized use of spontaneous imitation of both actions with objects and gestures, while facilitating gains in other social-communicative domains (Ingersoll and Schreibman, 2006).

The efficacy of RIT as an intervention for children with ASD is evidenced by multiple well-controlled research studies. Several experimental single-subject design experiments have demonstrate increases in object and gesture imitation, as well as highlighting gains in language and social skills as a result of RIT. For example, adopting a multiple-baseline design, Ingersoll and Schreibman (2006) found that after completion of the intervention phases, all five young children with ASD exhibited considerable improvements in object imitation, pretend play, joint attention and language. Importantly, such gains in imitation were found to generalize across materials, settings, and therapists (Ingersoll and Schreibman, 2006). Ingersoll and Gergans (2007) replicated these findings in a study investigating the effectiveness of parent-implemented RIT. Again, a multiple-baseline design across three families evidenced increased spontaneous object imitation in young children with autism, with effects exceeding the teaching period (Ingersoll and Gergans, 2007). Furthermore, in addition to object imitation, gains in gesture imitation have been demonstrated in a single-subject study by Ingersoll et al. (2007). In 2010, Ingersoll attempted to further validate these findings by conducting a pilot RCT into the effects of RIT on elicited and spontaneous imitation in autistic children (Ingersoll, 2010). Randomizing 21 young children into either RIT intervention or a control group, Ingersoll found larger imitation gains in the treatment group across all primary assessments, replicating previous single-subject findings. Thus, the large evidence-base for RIT as an effective intervention tool for autistic children is promising and, unlike other forms of ASD treatment, consists of multiple designs all demonstrating the same imitation, language, and social gains in this population.

Given the dynamic and effective nature of these play-based, reciprocal action and synchrony-oriented interventions, the ESDM and RIT appear to increase child-therapist social-motor synchrony (i.e., temporal coordination of movements) and social engagement (see also Landa et al., 2010). This increase in social-motor coordination and engagement may also increase social attention and motor resonance mechanisms in these children. Recall that there is evidence that activation of the MNS, FFA, and other social brain mechanisms may be limited in response to those individuals with whom children with autism are social-emotionally disconnected (e.g., unfamiliar people). Given that the ESDM and RIT increase social-communicative and language skills, one potential mechanistic pathway facilitating some of these behavioral changes is increased motor resonance through repeated social engagement with unfamiliar people. Evidence from a recent EEG/ERP study of face processing in toddlers with autism who received the ESDM vs. community-based services provides indirect support for this hypothesis. Specifically, the ESDM intervention increased late frontal activity in response to unfamiliar faces, relative to children who received TAU (Dawson et al., 2012). Because this was a study of static face processing, as opposed to human action processing, we cannot generalize these findings to the MNS without further research. However, direct experimental examinations of this hypothesis in the future, particularly experimental studies including measures of motor resonance, will be very informative in this regard.

## Discussion and future directions

We have described several interventions aimed at increasing social-communication and language skills in young children with autism that have theoretical and/or practical roots in relationships of these skills to motor development. In doing so, we have given serious consideration to the intervention methods as well as the existing or emerging evidence-base for each such intervention. As outlined in this review, neither practical nor theoretical links between motor and communication/language development are sufficient to predict the efficacy of an intervention for children on the autism spectrum. For example, despite very strong practical and theoretical links between early symbolic gestures, such as the iconic manual and motor signs of sign language, and speech and language development in typically developing children, extensive research suggests that SLT is not a very effective way to teach either communication or speech/language skills to children on the autism spectrum. On the other hand, evidence suggests that these children can learn a picture-based social-communication system, PECS, rapidly and effectively. Furthermore, research suggests that PECS is a relatively more effective path to speech development in these children. There are multiple potential reasons for this seemingly contradictory finding, including the possibility that impairments in motor skills (e.g., fine motor skills), motor imitation, and/or iconicity make learning and producing the manual and motor signs of sign language particularly challenging for children with ASDs (see also Figure 1 and Table 1).

We also reviewed and described several emerging intervention methods that take a more direct approach to motor aspects of speech production. These included PROMPT, which involves direct manipulations of the mouth and other sound-producing structures; AMMT, which aims to generate strong and direct temporal links between the child's auditory, motor, and speech production; and tDCS (and TMS), which involve directly stimulating motor and motor planning regions involved in speech production and other aspects of language. Although there is not yet existing experimental evidence for any of these interventions, reasonable pseudo-experimental/group case study reports on relatively well-characterized groups of children provide promising information to suggest that each of these interventions might prove effective for increase speech/language skills in this population. Therefore, experimental research is warranted on PROMPT, AMMT, and tDCS/TMS as potentially effective interventions for children with autism.

While the case study report on the group of minimally verbal children with autism receiving tDCS intervention is promising, there is also a need for caution in the pursuit of both research and practice involving the application of this technology to non-verbal or minimally verbal children with autism. While tDCS and TMS are generally believed to be safe procedures, there are also known risks (Wassermann, 1998; see also Loo et al., 2008; Rossi et al., 2009). For example, incorrect setting of electrical current or other parameters can trigger adverse events such as seizures, toxicity, headache, nausea, tissue damage, or burns. Furthermore, common adverse reactions include mild pain or sensitivity on the scalp, and headaches. It is, therefore, critically important to consider the ramifications involved with testing or treating non-verbal and minimally verbal children with autism with these technologies, given that they can neither provide informed consent nor effectively communicate injury or discomfort.

A risk of potentially even greater concern with the application of tDCS and TMS to children with autism is the potential for directly or indirectly causing seizure activity, or the onset of epilepsy. As characterized by Maski et al. (2011; see also Myers and Johnson, 2007), the prevalence of epilepsy is typically quoted in the literature as 30%. Identification of epilepsy in ASD is also challenging, due to the impact of ASD symptoms and behaviors on measurement/testing. As a result, assessing seizure risk would be very difficult to impossible for large numbers of non-verbal and minimally verbal children.

Despite the risks, tDCS and TMS have already been used to study children from a number of populations, including children who have experienced brain injury as a result of stroke (Frye et al., 2008; Kirton et al., 2010), children with language-learning disorders (Pugh et al., 2001), and children/adolescents with psychiatric disorders (Walter et al., 2001). Indeed, a clear strength of these technologies, and particularly tDCS, is that they are sufficiently streamlined and flexible in their application to be used with relatively young and relatively less able individuals. These techniques can even be used in conjunction with existing behavioral interventions (see Figure 3), potentially facilitating or enhancing their positive effects on speech and language development.

The possibility that the application of motor-related interventions might initiate the onset of even small to medium sized gains in speech development could have major long-term implications for quality of life. The results of several recent studies examining predictors of speech/language outcomes following early behavioral intervention suggest that a child producing even a few words prior to the start of intervention can play a key role in whether or not that child makes speech and language gains during the intervention (Gordon et al., 2011; Nahmias et al., 2012). Other research suggests that language abilities at 5- to 7-years of age are one of the key predictors of cognitive and adaptive skills outcomes in adulthood in this population (e.g., Gillespie-Lynch et al., 2012). At the same time, evidence suggests that relatively large percentages of autistic children who are completely non-verbal at 2-, 3-, and even 4-years of age develop speech and language skills fairly rapidly as a result of intensive early intervention (Koegel, 2000). Unfortunately, it is not currently possible to predict which non-verbal and minimally verbal young children will respond to any given early behavioral intervention (Stahmer et al., 2011).

As alluded to above, early intervention that targets speech and language skills by 2- to 4-years of age appear to be much more effective than those same interventions implemented after 5-years of age (Koegel, 2000), perhaps due to the existence of sensitive periods for speech/language and related skills (Fox et al., 2010; Windsor et al., 2011). Given the developmental complexity, and in some cases the seemingly strong biological nature, of motor development in relation to speech/language development, similar sensitive periods may exist in the relationships of motor and language/communication skills development. Therefore, the motor-related intervention pathways to language that have been discussed in this article, or others, may be most effective when intervention occurs in an ideal time window. Dependent upon the particular mechanism being targeted, this time window may be a sensitive biological/chronological age or developmental age period. For example, interventions that incorporate repetitive and coordinated hand banging may only be effective at facilitating speech when they occur during or shortly after the chronologically appropriate age of 7- to 12-months. Alternatively, intervening to increase these links may, as suggested by AMMT, still be effective at facilitating speech for any child below eight years of age who is in the pre-verbal or minimally verbal stage of development, for example. These are interesting clinical and empirical developmental questions, which can be directly examined in experimental studies.

## Conclusion

In this article, we have reviewed the research on aspects of early motor development that are believed to be specifically relevant to speech/language and social communication in infants and children with autism. We have also reviewed motor-related interventions designed to increase speech/language and social-communication skills in young non-verbal and minimally verbal children with autism. This field is at an exciting time in this area of research and development. We now know from extensive research that SLT is not a very effective intervention for facilitating speech and language development in this population. Potential reasons for this include that children with autism exhibit specific difficulties in iconicity, imitation of the actions of others, and/or fine motor skills, which make it difficult for them to become effective signers. On the other hand, these children appear to learn a picture-based social-communication program relatively rapidly, and extensive evidence suggests that this type of communication training does facilitate the development of basic speech skills in many of these children. At the same time as this, small-scale pseudo-experimental studies on at least three types of recently developed motor-based speech/language interventions (PROMPT, AMMT, tDCS/TMS) have each produced very promising results. This provides an exciting opportunity for important new experimental research studies designed to directly examine the efficacy of these interventions with this population, for whom effective speech/language interventions have been challenging to identify and develop. Finally, researchers with expertise in traditional applied behavior analytic and developmental interventions have begun working together to develop interventions that combine these two approaches. The result is a combined intervention strategy that uses highly effective operant teaching methods with a socially and motorically interactive play-based approach to enhancing speech/language and social-communication skills. The effects of these interventions on the children appear to extend beyond simple skill learning, and to enhance social attention and social engagement in ways that may facilitate the activation of social brain networks, including the motor-resonance system. We are optimistic that the field is approaching a turning point, with potentially dramatic breakthroughs to come in both our treatment and our understanding of the speech/language and social-communication difficulties in this population, as well as their relationship to motor mechanisms and development.

### Conflict of interest statement

The authors declare that the research was conducted in the absence of any commercial or financial relationships that could be construed as a potential conflict of interest.
